# Genomic Survey of *Bordetella pertussis* Diversity, United States, 2000–2013

**DOI:** 10.3201/eid2504.180812

**Published:** 2019-04

**Authors:** Michael R. Weigand, Margaret M. Williams, Yanhui Peng, Dane Kania, Lucia C. Pawloski, Maria L. Tondella, CDC Pertussis Working Group

**Affiliations:** Centers for Disease Control and Prevention, Atlanta, Georgia, USA

**Keywords:** pertussis, whooping cough, *Bordetella pertussis*, genomics, bacteria, United States, vaccines

## Abstract

We characterized 170 complete genome assemblies from clinical *Bordetella pertussis* isolates representing geographic and temporal diversity in the United States. These data capture genotypic shifts, including increased pertactin deficiency, occurring amid the current pertussis disease resurgence and provide a foundation for needed research to direct future public health control strategies.

Whooping cough (pertussis) remains a public health challenge in the United States where, despite high vaccine coverage, an increased number of cases have been reported since the late 1980s. This resurgence has included >48,000 cases reported in 2012 and notable recent statewide epidemics (*1*). Likely causes of the increase in reporting include heightened awareness, expanded surveillance, improved laboratory diagnostics, and waning protection conferred by acellular pertussis (aP) vaccine formulations (*1*,*2*).

The United States exclusively uses aP vaccines composed of inactivated *Bordetella pertussis* immunogenic proteins pertussis toxin (Pt), pertactin (Prn), and filamentous hemagglutinin (Fha), either with or without fimbria (Fim) types 2 and 3. Genetic divergence of circulating *B. pertussis* away from vaccine reference strains has led to allelic mismatch and the rapid emergence of Prn deficiency (*3*). Although such recent genetic changes may be ascribed to vaccine-driven immune selection (*4*), aP vaccines remain effective (*5*). 

The chromosome of *B. pertussis* also undergoes frequent structural rearrangement (*6*) that presents unique challenges to thorough investigation of genetic contributions to disease resurgence, limiting assessment of public health strategies. Until recently, genomic data with sufficient resolution to study sequence and structural variation were available only for vaccine and laboratory reference strains. However, pathogen evolution must be explored through multinomic characterization of circulating genotypes. To address this gap, we developed a dataset of complete, reference-quality genome sequence assemblies from isolates representing the geographic and temporal diversity of *B. pertussis* circulating in the United States during 2000–2013. 

## The Study

**Figure 1:**
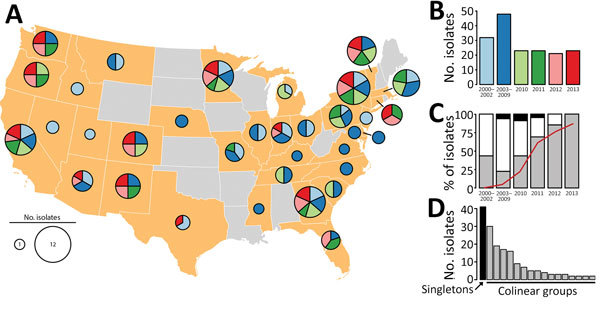
*Bordetella pertussis* diversity, United States, 2000–2013. A) Geographic origin of *B. pertussis* isolates selected to maximize the number of source states from each of 6 time periods. Pie chart diameter represents the number of isolates, as detailed in the key, and colors indicate time periods, as shown in panel B. B) Isolate frequency by time period. C) Relative abundance of MLST types *prn2-ptxP3-ptxA1-fimH1* (gray), *prn2-ptxP3-ptxA1-fimH2* (white), and other (black). Red line indicates frequency of pertactin-deficient alleles. D) Abundance distribution of genome structures. Black bar indicates unique structures (singletons) and gray bars the 16 colinear groups. MLST, multilocus sequence typing.

The Centers for Disease Control and Prevention (CDC) maintains a collection of *B. pertussis* isolates recovered by state public health laboratories through routine surveillance and outbreaks or the Enhanced Pertussis Surveillance/Emerging Infections Program Network (*7*). We selected a subset of isolates (n = 170) to account for potential geographic diversity. We stratified all isolates in the collection by state and time period (2000–2002, 2003–2009, 2010, 2011, 2012, and 2013) chosen according to diversity indices reported previously (*8*), with additional emphasis on more recent sampling. We then randomly sampled the stratified collection to maximize the number of source states (n = 34) during each period with equal weighting (Figure 1, panel A, B). Most isolates were characterized by existing molecular approaches, multilocus variable-number tandem-repeat analysis (MLVA), and pulsed-field gel electrophoresis (PFGE), as described previously (*9*). The selected isolates included 17 MLVA types, with type 27 the most prevalent, and 33 PFGE profiles, with profile CDC013 the most prevalent (Appendix Table 1).

We performed whole-genome shotgun sequencing and assembly as described previously (*10*) (Appendix). Genome assembly yielded a single circular contig for all isolates, and we performed sequence-based molecular typing (Appendix). Nearly all isolates (96%) were of the predominant type *prn2-ptxP3-ptxA1* with either *fimH1* or *fimH2*, and few harbored alternate types such as *prn1-ptxP1-ptxA2-fimH1* (Figure 1, panel C). Prn deficiency has been observed in >16 independent mutations to *prn* (*6*); we observed 10 deficient alleles among 57/170 isolates in our study, including missense substitutions, deletions, promoter disruption, and various IS*481* insertions. The proportion of isolates with Prn-deficient alleles increased rapidly beginning in 2010 (Figure 1, panel C), consistent with a larger molecular survey of US isolates conducted previously that included some used in this study (*3*). We also determined MLVA type from genome assemblies using a custom bioinformatics pipeline (wgsMLVA) based on traditional PCR primer sequences (*11*) (Appendix). None of the genomes encoded known 23S ribosomal RNA mutation associated with erythromycin resistance (*12*).

To determine variation in chromosome structure, we performed exhaustive pairwise alignment of assembled genomes as previously described (*6*). Of the 170 assemblies, 129 clustered into 16 groups of >2 colinear genomes (lacking observable rearrangement or deletion >1,500 bp), whereas 41 assemblies (singletons) exhibited unique structures not shared with any others in the dataset. Observed structures largely correlated with PFGE, a proxy for chromosome structure, clustering isolates with shared PFGE profiles. The abundance of common structures reflected predominant PFGE profiles, and the largest cluster corresponded to profile CDC013 (Figure 1, panel D). Differences between many common structures could be attributed to large inversions flanked by insertions of the multicopy IS*481*. Select singleton structures resulted from tandem duplication of large regions (15.5–190 kbp) in the genomes of 5 isolates (D236, D665, H624, J085, and J139) that were also flanked by copies of IS*481*.

**Figure 2:**
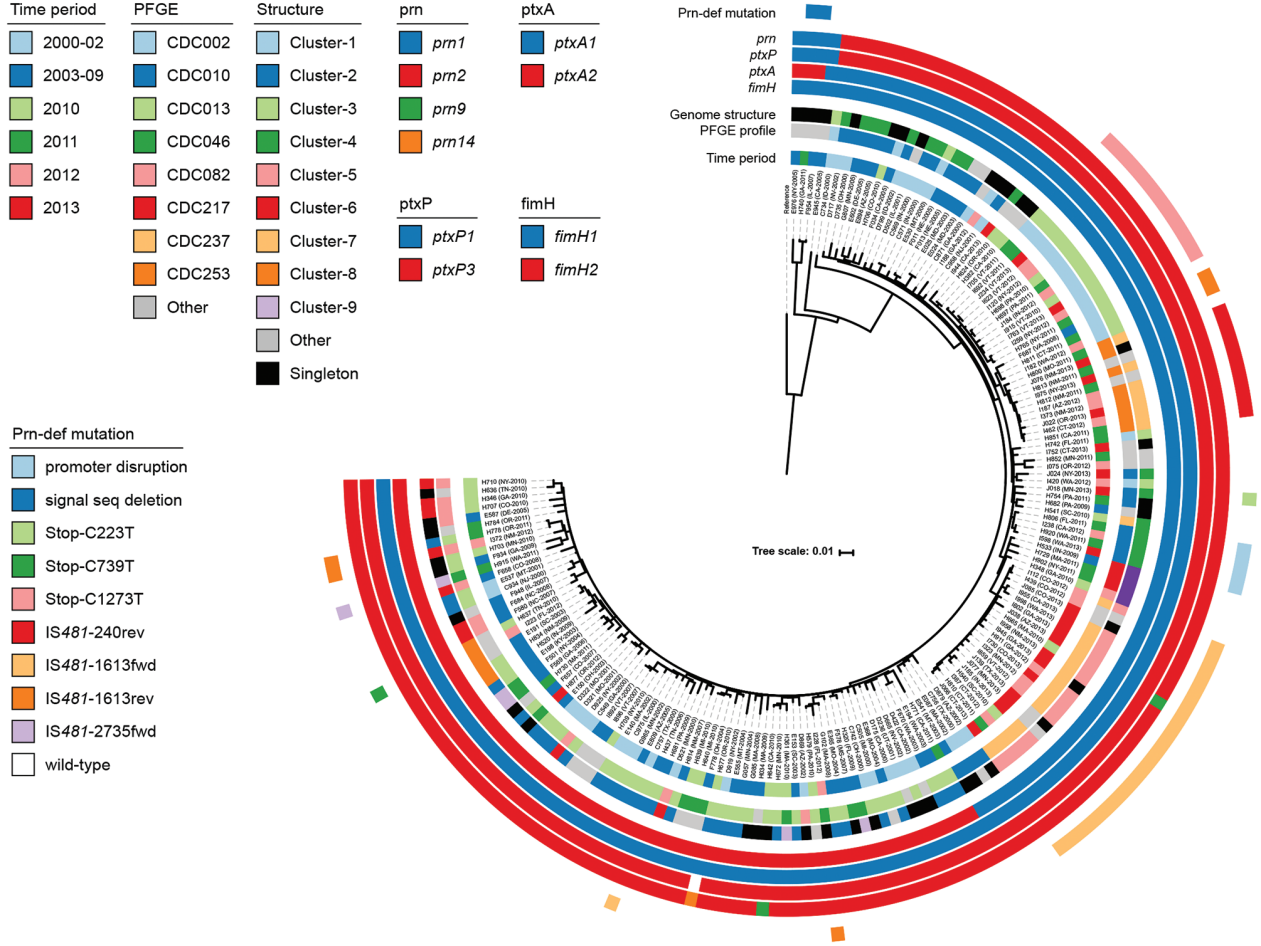
Phylogenetic reconstruction of all 170 isolates and the reference Tohama I (GenBank accession no. CP010964). Isolate metadata and molecular characteristics are color coded, as detailed in the key. Scale bar indicates substitutions per site. CDC, Centers for Disease Control and Prevention; fim, fimbria; fwd, forward insertion; rev, reverse insertion; PFGE, pulsed-field gel electrophoresis; prn, pertactin; ptx, pertussis toxin.

We reconstructed a maximum-likelihood phylogeny of the isolate genomes from 840 core variable single-nucleotide polymorphisms (SNPs) determined from the reference Tohama I (GenBank accession no. CP010964) (Appendix). The resulting tree topology revealed deep divergence of lineages bearing alleles *ptxP1* and *ptxP3*, as well as clear distinctions between clades of *prn2-ptxP3-ptxA1-fimH1* and *prn2-ptxP3-ptxA1-fimH2* (Figure 2). Only certain *prn*-disrupting mutations (e.g., nonsense C1273T, promoter disruption) and chromosome structures (e.g., cluster-4, cluster-6, cluster-7, cluster-9) appeared phylogenetically linked, meaning isolates sharing them were also related according to their SNP patterns. However, each group of related isolates was recovered across multiple states and time periods, suggesting that genotypes, whether defined by gene sequence or chromosome structure, were stable enough to be widely circulated. Prn deficiency due to IS*481* disruption has resulted from >7 independent events among the isolates in this dataset, but related isolates with these mutations were likewise geographically and temporally distributed. These results are consistent with phylogenies of circulating *B. pertussis* reported elsewhere (*6*,*13*).

## Conclusions

We have developed a representative dataset of complete genome sequence assemblies derived from *B. pertussis* clinical isolates recovered in the United States that captures shifting population genetics concurrent with disease resurgence. We selected isolates to maximize the geographic diversity of circulating *B. pertussis* across 6 time periods during 2000–2013 and to span the time period in which Prn deficiency emerged as the predominant molecular type. Although the sparse sampling of individual states and regions prohibited detailed analyses of geographic distribution, we did recover isolates with shared SNP patterns and chromosome structures from disparate states. Our results illustrate underlying challenges to the molecular study of pertussis resurgence, including a circulating mixture of gene sequence (SNP) and chromosome structure variants.

The genomic data we provide will aid open research toward improved vaccine development and disease control strategies. Because little to no such high-quality data existed previously, the contribution of genome evolution to pertussis resurgence has not been fully appreciated. A subset of these data has already helped elucidate historical patterns of chromosome rearrangement (*6*). However, comparative genomics alone is not sufficient to understand the resurgence in pertussis. Further laboratory experimentation using in vitro and in vivo infection models is needed to link outcomes with novel, bioinformatically determined genetic variation, such as discrete rearrangements and tandem duplications. Potential differences in antigen expression resulting from these changes in gene organization, which may influence the burden of disease, remain untested. Our results provide needed context to guide such investigations by highlighting representative, circulating genotypes as they continue their divergence from existing laboratory and vaccine reference strains. Data such as those presented here critically establish the necessary foundation for collaborative development of advanced diagnostics, novel molecular typing methods, and improved vaccine formulations.

Appendix Additional information about *Bordetella pertussis* diversity in the United States.
