# Supplementary material for: Genomic Survey of Bordetella pertussis Diversity, United States, 2000–2013
Source: Emerg Infect Dis. 2019 Apr;25(4):780–3. doi: 10.3201/eid2504.180812 (PMC6433035; doi:10.3201/eid2504.180812)
Supplement: Appendix — Additional information about Bordetella pertussis diversity in the United States. [file 18-0812-Techapp-s1.pdf]

Article DOI: https://doi.org/10.3201/eid2504.180812

Genomic Survey of *Bordetella pertussis* Diversity in the United States, 2000–2013

Appendix

Methods

We performed whole-genome shotgun sequencing using a combination of the PacBio RSII (Pacific Biosciences, http://www.pacb.com/), Illumina HiSeq/MiSeq (Illumina, http://www.illumina.com/), and Argus (OpGen; http://www.opgen.com) platforms as described previously (*1*). The cumulative coverage depth of PacBio and Illumina sequencing for each isolate is listed in Appendix Table 1. Genomes were assembled using HGAP v3 (Pacific Biosciences) followed by structure confirmation with restriction digest optical mapping (OpGen) and further sequence polishing by Illumina read mapping with CLC Genomics Workbench (QIAGEN, http://www.qiagen.com). Completed assemblies were submitted to the National Center for Biotechnology Information (NCBI) for annotation by the automated Prokaryotic Genome Annotation Pipeline (PGAP). Genome sequence-based molecular characterization was performed using either completed assemblies or individual sequencing reads. Alleles for common molecular typing loci (*ptxP*, *ptxA*, *ptxB*, *fimH*, and *prn*) were assigned by genome alignment to a curated set of wild-type and deficient alleles using high-stringency.

Molecular typing by multiple-locus variable number tandem repeat analysis (MLVA) was determined from closed genome assemblies using a custom bioinformatics pipeline (wgsMLVA). Similar to traditional PCR-based approaches, wgsMLVA uses primer sequences to identify 6 Variable Number Tandem Repeat (VNTR) sites which contain a varying set of short sequence repeats (*2*). The number of repeat monomers is counted at each site to build a 6-number VNTR profile summarized as an MLVA type (www.mlva.net). Traditionally, each VNTR is amplified by PCR and repeat numbers are inferred from the molecular weight; with higher molecular weights corresponding to the addition of known repeat monomers. By contrast, wgsMLVA leverages high resolution genome assemblies to directly count repeat monomers in

each VNTR site using an exact-match search. This approach produces a more accurate count that does not rely on estimations calculated from VNTR length.

Of the 170 isolates characterized in this study, 128 had been characterized by traditional MLVA using PCR before whole-genome sequencing. Profiles calculated using wgsMLVA were identical to those determined by traditional MLVA for 127/128 (99.2%) isolates (Appendix Table 1). Comparison of VNTR profiles in H811 calculated by the 2 methods revealed a discrepancy of 1 repeat in a VNTR3 locus (Appendix Table 2). Traditional MLVA cannot differentiate the 2 VNTR3 loci and relies on measurable differences in electrophoretic mobility; otherwise, VNTR3b is counted as 0 ambiguously reporting it as either missing or equal to VNTR3a. Because wgsMLVA directly counts repeat monomers at each locus independently, such a discrepancy is not wholly unexpected given that VNTR3 encodes the smallest repeat monomer at 5 bp, compared with the larger 15 bp monomer of VNTR1.

The wgsMLVA pipeline is implemented in Python 2.7, free of external libraries, packages, or other dependencies. A user-supplied reference database is required to match MLVA types from calculated VNTR profiles; an updated database can be downloaded from www.mlva.net.

Single nucleotide polymorphisms in each isolate genome were determined from the reference Tohama I (CP010964) by mapping Illumina reads with snippy (https://github.com/tseemann/snippy). A maximum-likelihood phylogeny was reconstructed using RAxML (*3*) and additional tree annotation was performed using iTOL (*4*).

Source Code

The source code for calculating MLVA types from complete genome assemblies of *Bordetella pertussis* using wgsMLVA is available at https://github.com/danek90/wgsMLVA

References

1. Bowden KE, Weigand MR, Peng Y, Cassiday PK, Sammons S, Knipe K, et al. Genome structural diversity among 31 *Bordetella pertussis* isolates from two recent US whooping cough statewide epidemics. mSphere. 2016; 1:e00036–16. http://dx.doi.org/10.1128/mSphere.00036-16

2. Schouls LM, van der Heide HG, Vauterin L, Vauterin P, Mooi FR. Multiple-locus variable-number tandem repeat analysis of Dutch *Bordetella pertussis* strains reveals rapid genetic changes with clonal expansion during the late 1990s. J Bacteriol. 2004;186:5496–505. PubMed http://dx.doi.org/10.1128/JB.186.16.5496-5505.2004

3. Stamatakis A. RAxML version 8: a tool for phylogenetic analysis and post-analysis of large phylogenies. Bioinformatics. 2014;30:1312–3. PubMed http://dx.doi.org/10.1093/bioinformatics/btu033

4. Letunic I, Bork P. Interactive tree of life (iTOL) v3: an online tool for the display and annotation of phylogenetic and other trees. Nucleic Acids Res. 2016;44(W1):W242–5. PubMed http://dx.doi.org/10.1093/nar/gkw290

5. Weigand MR, Peng Y, Loparev V, Batra D, Bowden KE, Burroughs M, et al. The history of *Bordetella pertussis* genome evolution includes structural rearrangement. J Bacteriol. 2017;199:e00806–16. PubMed http://dx.doi.org/10.1128/JB.00806-16

Appendix Table 1. Detailed data about *Bordetella pertussis* isolates, 2000–2013*

ID	Year	State	EPS†	MLVA	wgsMLVA	PFGE	Structure	Molecular typing loci *prn*	*ptxP*	*ptxA*	*fimH*	Accession no.	Depth‡	Reference
C505	2000	MI		NT	27	CDC013	Cluster-1	*prn2*	*ptxP3*	*ptxA1*	*fimH2*	CP011687	293x	5
C549	2000	GA		36	36	CDC013	Cluster-1	*prn2*	*ptxP3*	*ptxA1*	*fimH2*	CP013077	311x	5
C569	2000	IN		27	27	CDC010	Cluster-3	*prn2*	*ptxP3*	*ptxA1*	*fimH1*	CP025347	382x	This study
C571	2000	IN		27	27	CDC010	Cluster-3	*prn2*	*ptxP3*	*ptxA1*	*fimH1*	CP011167	265x	5
C734	2000	ID		27	27	CDC002	Cluster-2	*prn2*	*ptxP3*	*ptxA1*	*fimH1*	CP013078	278x	5
C742	2000	OH		NT	27	CDC013	Singleton	*prn2*	*ptxP3*	*ptxA1*	*fimH2*	CP011688	254x	5
C756	2000	TX		NT	27	CDC010	Cluster-10	*prn2*	*ptxP3*	*ptxA1*	*fimH1*	CP025368	337x	This study
C757	2000	TX		16	16	CDC013	Cluster-1	*prn2*	*ptxP3*	*ptxA1*	*fimH2*	CP013079	292x	5
C871	2000	GA		NT	27	CDC007	Singleton	*prn2*	*ptxP3*	*ptxA1*	*fimH1*	CP025345	413x	This study
C934	2000	NJ		27	27	CDC013	Cluster-1	*prn2*	*ptxP3*	*ptxA1*	*fimH2*	CP016961	343x	This study
C958	2001	NJ		27	27	CDC010	Singleton	*prn2*	*ptxP3*	*ptxA1*	*fimH1*	CP011168	349x	5
C975	2000	IL		NT	38	CDC013	Cluster-1	*prn2*	*ptxP3*	*ptxA1*	*fimH2*	CP013868	377x	5
D175	2000	CA		NT	200	CDC013	Cluster-1	*prn2*	*ptxP3*	*ptxA1*	*fimH2*	CP011689	262x	5
D236	2001	UT		27	27	CDC150	Singleton	*prn2*	*ptxP3*	*ptxA1*	*fimH2*	CP025530	284x	This study
D321	2001	MO		27	27	CDC013	Cluster-1	*prn2*	*ptxP3*	*ptxA1*	*fimH2*	CP011690	290x	5
D322	2001	MO		27	27	CDC013	Cluster-1	*prn2*	*ptxP3*	*ptxA1*	*fimH2*	CP025358	337x	This study
D422	2002	CA		18	18	CDC154	Singleton	*prn2*	*ptxP3*	*ptxA1*	*fimH2*	CP016959	338x	This study
D502	2001	IL		NT	27	CDC007	Singleton	*prn2*	*ptxP3*	*ptxA1*	*fimH1*	CP011691	293x	5
D521	2000	MN		NT	27	CDC082	Cluster-5	*prn2*	*ptxP3*	*ptxA1*	*fimH2*	CP011169	261x	5
D665	2002	NV		27	27	CDC013	Singleton	*prn2*	*ptxP3*	*ptxA1*	*fimH2*	CP025526	258x	This study
D717	2002	NV		27	27	CDC010	Cluster-3	*prn2*	*ptxP3*	*ptxA1*	*fimH1*	CP016964	384x	This study
D735	2000	OH		NT	18	CDC010	Singleton	*prn2*	*ptxP3*	*ptxA1*	*fimH1*	CP016960	399x	This study
D799	2002	ID		27	27	CDC010	Cluster-3	*prn2*	*ptxP3*	*ptxA1*	*fimH1*	CP016963	414x	This study
D869	2002	AZ		NT	25	CDC082	Singleton	*prn2*	*ptxP3*	*ptxA1*	*fimH2*	CP025528	233x	This study
D879	2002	AZ		NT	36	CDC010	Singleton	*prn2*	*ptxP3*	*ptxA1*	*fimH1*	CP011170	234x	5
D919	2002	NY		NT	27	CDC013	Cluster-1	*prn2*	*ptxP3*	*ptxA1*	*fimH2*	CP025355	219x	This study
D925	2002	NY		27	27	CDC082	Cluster-5	*prn2*	*ptxP3*	*ptxA1*	*fimH2*	CP016968	369x	This study
E024	2003	MD		27	27	CDC010	Cluster-10	*prn2*	*ptxP3*	*ptxA1*	*fimH1*	CP011692	236x	5
E025	2003	MD		27	27	CDC010	Cluster-10	*prn2*	*ptxP3*	*ptxA1*	*fimH1*	CP016967	320x	This study
E087	2002	MA		NT	27	CDC010	Singleton	*prn2*	*ptxP3*	*ptxA1*	*fimH1*	CP025480	295x	This study
E140	2002	MA		NT	16	CDC013	Cluster-1	*prn2*	*ptxP3*	*ptxA1*	*fimH2*	CP025354	420x	This study
E150	2003	OH		32	32	CDC013	Cluster-1	*prn2*	*ptxP3*	*ptxA1*	*fimH2*	CP011171	327x	5
E153	2003	SC		NT	28	CDC013	Cluster-1	*prn2*	*ptxP3*	*ptxA1*	*fimH2*	CP025359	382x	This study
E191	2003	SC		27	27	CDC123	Cluster-7	*prn2*	*ptxP3*	*ptxA1*	*fimH2*	CP025478	252x	This study
E194	2003	WA		27	27	CDC013	Cluster-1	*prn2*	*ptxP3*	*ptxA1*	*fimH2*	CP013080	353x	5
E198	2003	KY		26	26	CDC013	Cluster-1	*prn2*	*ptxP3*	*ptxA1*	*fimH2*	CP025385	203x	This study
E365	2004	MO		27	27	CDC013	Cluster-1	*prn2*	*ptxP3*	*ptxA1*	*fimH2*	CP025387	309x	This study
E368	2004	MO		25	25	CDC013	Cluster-13	*prn2*	*ptxP3*	*ptxA1*	*fimH2*	CP013869	430x	5
E530	2000	MT		NT	27	CDC002	Cluster-2	*prn2*	*ptxP3*	*ptxA1*	*fimH1*	CP011693	200x	5
E537	2001	MT		27	27	CDC013	Cluster-1	*prn2*	*ptxP3*	*ptxA1*	*fimH2*	CP016958	352x	This study
E541	2003	MT		27	27	CDC010	Singleton	*prn2*	*ptxP3*	*ptxA1*	*fimH1*	CP016966	403x	This study
E555	2004	MT		NT	27	CDC013	Cluster-1	*prn2*	*ptxP3*	*ptxA1*	*fimH2*	CP011172	332x	5
E587	2005	DE		27	27	CDC082	Singleton	*prn2*	*ptxP3*	*ptxA1*	*fimH2*	CP011173	309x	5
E602	2005	DE		218	218	CDC010	Cluster-3	*prn2*	*ptxP3*	*ptxA1*	*fimH1*	CP013081	348x	5
E809	2005	AZ		NT	18	CDC013	Cluster-1	*prn2*	*ptxP3*	*ptxA1*	*fimH2*	CP011174	305x	5

								Molecular typing loci						
ID	Year	State	EPS†	MLVA	wgsMLVA	PFGE	Structure	*prn*	*ptxP*	*ptxA*	*fimH*	Accession no.	Depth‡	Reference
E898	2005	AZ		16	16	CDC010	Cluster-3	*prn2*	*ptxP3*	*ptxA1*	*fimH1*	CP016962	297x	This study
E945	2005	CA		70	70	CDC021	Singleton	*prn1*	*ptxP1*	*ptxA1*	*fimH1*	CP016956	365x	This study
E976	2005	NY		NT	227	CDC020	Singleton	*prn1*	*ptxP1*	*ptxA2*	*fimH1*	CP011175	267x	5
F011	2005	NE		27	27	CDC010	Cluster-3	*prn2*	*ptxP3*	*ptxA1*	*fimH1*	CP011176	278x	5
F013	2005	NE		27	27	CDC010	Cluster-3	*prn2*	*ptxP3*	*ptxA1*	*fimH1*	CP016965	361x	This study
F034	2005	CA		NT	27	CDC002	Singleton	*prn2*	*ptxP3*	*ptxA1*	*fimH1*	CP011177	288x	5
F501	2004	NY		NT	27	CDC046	Cluster-8	*prn2*	*ptxP3*	*ptxA1*	*fimH2*	CP013870	320x	5
F569	2006	GA		NT	27	CDC046	Cluster-8	*prn2*	*ptxP3*	*ptxA1*	*fimH2*	CP025523	370x	This study
F578	2007	MS		27	27	CDC046	Singleton	*prn2*	*ptxP3*	*ptxA1*	*fimH2*	CP025357	400x	This study
F580	2007	NC		27	27	CDC082	Cluster-5	*prn2*	*ptxP3*	*ptxA1*	*fimH2*	CP025342	505x	This study
F657	2007	CO		NT	179	CDC013	Cluster-1	*prn2*	*ptxP3*	*ptxA1*	*fimH2*	CP013871	440x	5
F658	2008	CO		176	176	CDC082	Cluster-5	*prn2*	*ptxP3*	*ptxA1*	*fimH2*	CP011178	302x	5
F670	2003	WA		27	27	CDC013	Cluster-1	*prn2*	*ptxP3*	*ptxA1*	*fimH2*	CP011179	305x	5
F684	2008	NC		36	36	CDC082	Cluster-5	*prn2*	*ptxP3*	*ptxA1*	*fimH2*	CP011180	392x	5
F687	2008	VA		27	27	CDC002	Cluster-2	*prn2*	*ptxP3*	*ptxA1*	*fimH1*	CP011181	275x	5
F778	2004	OH		27	27	CDC046	Cluster-11	*prn2*	*ptxP3*	*ptxA1*	*fimH2*	CP013872	527x	5
F934	2009	GA		NT	27	CDC013	Singleton	*prn2*	*ptxP3*	*ptxA1*	*fimH2*	CP013873	410x	5
F948	2007	IL		NT	158	CDC171	Singleton	*prn2*	*ptxP3*	*ptxA1*	*fimH2*	CP011182	388x	5
F954	2007	IL		186	186	CDC260	Singleton	*prn1*-signal_seq_del	*ptxP1*	*ptxA2*	*fimH1*	CP025366	338x	This study
G057	2004	MN		NT	26	CDC013	Cluster-1	*prn2*	*ptxP3*	*ptxA1*	*fimH2*	CP012129	369x	5
G085	2008	MA		NT	16	CDC013	Singleton	*prn2*	*ptxP3*	*ptxA1*	*fimH2*	CP013874	416x	5
G102	2008	MA		NT	27	CDC013	Cluster-1	*prn2*	*ptxP3*	*ptxA1*	*fimH2*	CP025388	248x	This study
G807	2005	MN		NT	27	CDC010	Cluster-3	*prn2*	*ptxP3*	*ptxA1*	*fimH1*	CP013875	383x	5
G965	2002	MN		NT	16	CDC013	Cluster-1	*prn2*	*ptxP3*	*ptxA1*	*fimH2*	CP013876	406x	5
H034	2009	MA		NT	27	CDC242	Singleton	*prn2*	*ptxP3*	*ptxA1*	*fimH2*	CP025356	322x	This study
H320	2009	FL		27	27	CDC046	Cluster-8	*prn2*	*ptxP3*	*ptxA1*	*fimH2*	CP011234	407x	5
H346	2010	GA		158	158	CDC082	Cluster-5	*prn2*	*ptxP3*	*ptxA1*	*fimH2*	CP011694	236x	5
H348	2010	GA		27	27	CDC082	Cluster-9	*prn2*	*ptxP3*	*ptxA1*	*fimH1*	CP013877	366x	5
H361	2010	MA		27	27	CDC046	Cluster-8	*prn2*	*ptxP3*	*ptxA1*	*fimH2*	CP013878	224x	5
H382	2010	CA		27	27	CDC270	Singleton	*prn2*	*ptxP3*	*ptxA1*	*fimH1*	CP013082	334x	5
H437	2006	TN		NT	77	CDC013	Cluster-1	*prn2*	*ptxP3*	*ptxA1*	*fimH2*	CP011695	258x	5
H520	2009	IN		NT	27	CDC013	Cluster-7	*prn2*	*ptxP3*	*ptxA1*	*fimH2*	CP011183	249x	5
H533	2009	IN		NT	N/A	CDC217	Cluster-9	*prn2*	*ptxP3*	*ptxA1*	*fimH1*	CP013879	288x	5
H540	2010	SC		NT	28	CDC237	Cluster-4	*prn2*-IS*481*-1613fwd	*ptxP3*	*ptxA1*	*fimH1*	CP013880	292x	5
H541	2010	SC		NT	27	CDC237	Cluster-3	*prn2*	*ptxP3*	*ptxA1*	*fimH1*	CP025373	266x	This study
H579	2010	PA		128	128	CDC013	Cluster-13	*prn2*	*ptxP3*	*ptxA1*	*fimH2*	CP011184	336x	5
H624	2010	OR	Y	27	27	CDC270	Singleton	*prn2*	*ptxP3*	*ptxA1*	*fimH1*	CP025529	391x	This study
H636	2010	TN		16	16	CDC278	Singleton	*prn2*-wt-C638T	*ptxP3*	*ptxA1*	*fimH2*	CP013881	249x	5
H637	2010	TN		NT	27	CDC123	Cluster-7	*prn2*-Stop-C739T	*ptxP3*	*ptxA1*	*fimH2*	CP011185	270x	5
H639	2010	MI		36	36	CDC046	Cluster-11	*prn2*	*ptxP3*	*ptxA1*	*fimH2*	CP012130	209x	5
H640	2010	MI		36	36	CDC046	Cluster-11	*prn2*	*ptxP3*	*ptxA1*	*fimH2*	CP025371	406x	This study
H642	2010	CA		27	27	CDC013	Singleton	*prn9*	*ptxP3*	*ptxA1*	*fimH2*	CP025360	427x	This study
H665	2010	MA	Y	27	27	CDC237	Cluster-4	*prn2*-IS*481*-1613fwd	*ptxP3*	*ptxA1*	*fimH1*	CP011186	342x	5
H672	2010	MN	Y	27	27	CDC013	Cluster-1	*prn2*	*ptxP3*	*ptxA1*	*fimH2*	CP025349	443x	This study
H677	2010	OR		27	27	CDC013	Cluster-1	*prn14*	*UNK*	*ptxA1*	*fimH2*	CP025367	417x	This study
H681	2009	PA		NT	27	CDC013	Cluster-1	*prn2*-IS*481*-1613fwd	*ptxP3*	*ptxA1*	*fimH2*	CP012078	335x	5
H682	2009	PA		NT	18	CDC125	Singleton	*prn2*	*ptxP3*	*ptxA1*	*fimH1*	CP013083	260x	5

								Molecular typing loci						
ID	Year	State	EPS†	MLVA	wgsMLVA	PFGE	Structure	*prn*	*ptxP*	*ptxA*	*fimH*	Accession no.	Depth‡	Reference
H697	2011	PA		NT	27	CDC002	Cluster-2	*prn2*-Stop-C1273T	*ptxP3*	*ptxA1*	*fimH1*	CP025365	375x	This study
H698	2010	PA		27	27	CDC002	Cluster-2	*prn2*-Stop-C1273T	*ptxP3*	*ptxA1*	*fimH1*	CP013084	366x	5
H703	2010	MN		27	27	CDC013	Singleton	*prn2*	*ptxP3*	*ptxA1*	*fimH2*	CP011187	220x	5
H706	2010	CO		27	27	CDC010	Singleton	*prn2*	*ptxP3*	*ptxA1*	*fimH1*	CP013085	372x	5
H707	2010	CO		120	120	CDC082	Cluster-5	*prn2*	*ptxP3*	*ptxA1*	*fimH2*	CP011188	279x	5
H709	2010	NY		16	16	CDC037	Cluster-12	*prn2*	*ptxP3*	*ptxA1*	*fimH2*	CP025364	370x	This study
H710	2010	NY		16	16	CDC082	Cluster-5	*prn2*-wt-C638T	*ptxP3*	*ptxA1*	*fimH2*	CP011236	329x	5
H729	2011	MA		27	27	CDC217	Cluster-9	*prn2*	*ptxP3*	*ptxA1*	*fimH1*	CP011189	239x	5
H730	2011	MA		22	22	CDC202	Singleton	*prn2*	*ptxP3*	*ptxA1*	*fimH2*	CP013086	165x	5
H740	2011	GA		NT	186	CDC266	Singleton	*prn1*-signal_seq_del	*ptxP1*	*ptxA2*	*fimH1*	CP011190	188x	5
H742	2011	FL		27	27	CDC002	Cluster-2	*prn2*	*ptxP3*	*ptxA1*	*fimH1*	CP025346	195x	This study
H754	2011	PA		NT	27	CDC010	Singleton	*prn2*	*ptxP3*	*ptxA1*	*fimH1*	CP011191	284x	5
H765	2011	NY		27	27	CDC002	Cluster-2	*prn2*-Stop-C1273T	*ptxP3*	*ptxA1*	*fimH1*	CP011192	259x	5
H771	2011	CA		27	27	CDC013	Cluster-1	*prn2*	*ptxP3*	*ptxA1*	*fimH2*	CP013087	107x	5
H778	2011	OR	Y	27	27	CDC013	Cluster-1	*prn2*-IS*481*-1613rev	*ptxP3*	*ptxA1*	*fimH2*	CP025362	207x	This study
H784	2011	OR	Y	27	27	CDC273	Singleton	*prn2*	*ptxP3*	*ptxA1*	*fimH2*	CP011193	230x	5
H800	2011	MO		27	27	CDC253	Cluster-6	*prn2*	*ptxP3*	*ptxA1*	*fimH1*	CP011194	268x	5
H806	2011	FL		27	27	CDC010	Cluster-3	*prn2*-promoter_disrupt	*ptxP3*	*ptxA1*	*fimH1*	CP011195	304x	5
H810	2011	CT	Y	27	27	CDC237	Cluster-4	*prn2*-IS*481*-1613fwd	*ptxP3*	*ptxA1*	*fimH1*	CP011196	435x	5
H811	2011	CT	Y	27	18	CDC002	Cluster-2	*prn2*-IS*481*-1613rev	*ptxP3*	*ptxA1*	*fimH1*	CP025361	453x	This study
H812	2011	NM	Y	27	27	CDC269	Cluster-14	*prn2*-IS*481*-240rev	*ptxP3*	*ptxA1*	*fimH1*	CP011197	256x	5
H813	2011	NM	Y	27	27	CDC269	Cluster-14	*prn2*-IS*481*-240rev	*ptxP3*	*ptxA1*	*fimH1*	CP025351	371x	This study
H814	2007	NM		27	27	CDC013	Cluster-1	*prn2*	*ptxP3*	*ptxA1*	*fimH2*	CP025374	412x	This study
H834	2009	NM		27	27	CDC013	Cluster-7	*prn2*	*ptxP3*	*ptxA1*	*fimH2*	CP011235	205x	5
H851	2011	CA		27	27	CDC253	Cluster-6	*prn2*-IS*481*-240rev	*ptxP3*	*ptxA1*	*fimH1*	CP011237	250x	5
H852	2011	MN	Y	27	27	CDC024	Cluster-15	*prn2*	*ptxP3*	*ptxA1*	*fimH1*	CP012079	262x	5
H877	2012	OR	Y	32	32	CDC046	Singleton	*prn2*	*ptxP3*	*ptxA1*	*fimH2*	CP025382	388x	This study
H902	2011	NY		36	36	CDC217	Cluster-9	*prn2*	*ptxP3*	*ptxA1*	*fimH1*	CP025363	434x	This study
H911	2012	GA		27	27	CDC237	Cluster-4	*prn2*-IS*481*-1613fwd	*ptxP3*	*ptxA1*	*fimH1*	CP011238	260x	5
H915	2011	WA		27	27	CDC046	Cluster-8	*prn2*-IS*481*-2735fwd	*ptxP3*	*ptxA1*	*fimH2*	CP011239	273x	5
H920	2011	WA		27	27	CDC010	Cluster-3	*prn2*-promoter_dis	*ptxP3*	*ptxA1*	*fimH1*	CP025352	433x	This study
I075	2012	OR	Y	27	27	CDC326	Cluster-15	*prn2*	*ptxP3*	*ptxA1*	*fimH1*	CP011240	422x	5
I112	2012	CO	Y	27	27	CDC237	Cluster-4	*prn2*-IS*481*-1613fwd	*ptxP3*	*ptxA1*	*fimH1*	CP011241	248x	5
I120	2012	NY	Y	27	27	CDC002	Cluster-2	*prn2*-Stop-C1273T	*ptxP3*	*ptxA1*	*fimH1*	CP025370	377x	This study
I182	2012	WA		158	158	CDC002	Cluster-2	*prn2*-IS*481*-1613rev	*ptxP3*	*ptxA1*	*fimH1*	CP026996	176x	This study
I187	2012	AZ		27	27	CDC253	Cluster-6	*prn2*-IS*481*-240rev	*ptxP3*	*ptxA1*	*fimH1*	CP012132	141x	5
I188	2012	GA		27	27	CDC002	Singleton	*prn2*	*ptxP3*	*ptxA1*	*fimH1*	CP025379	393x	This study
I223	2012	FL		27	27	CDC123	Cluster-7	*prn2*	*ptxP3*	*ptxA1*	*fimH2*	CP025369	336x	This study
I228	2012	FL		27	27	CDC046	Singleton	*prn2*-IS*481*-1613rev	*ptxP3*	*ptxA1*	*fimH2*	CP011198	334x	5
I238	2012	CA		27	27	CDC010	Cluster-3	*prn2*-promoter_dis	*ptxP3*	*ptxA1*	*fimH1*	CP011199	248x	5
I259	2012	NY	Y	27	27	CDC002	Cluster-2	*prn2*-Stop-C1273T	*ptxP3*	*ptxA1*	*fimH1*	CP012133	183x	5
I323	2012	MN	Y	27	27	CDC237	Cluster-4	*prn2*-IS*481*-1613fwd	*ptxP3*	*ptxA1*	*fimH1*	CP025377	365x	This study
I372	2012	NM	Y	27	27	CDC082	Cluster-5	*prn2*-IS*481*-1613rev	*ptxP3*	*ptxA1*	*fimH2*	CP025372	358x	This study
I373	2012	NM	Y	27	27	CDC253	Cluster-6	*prn2*-IS*481*-240rev	*ptxP3*	*ptxA1*	*fimH1*	CP011200	292x	5
I387	2012	CT	Y	27	27	CDC237	Cluster-4	*prn2*-IS*481*-1613fwd	*ptxP3*	*ptxA1*	*fimH1*	CP011201	425x	5
I420	2012	WA		27	27	CDC002	Cluster-2	*prn2*	*ptxP3*	*ptxA1*	*fimH1*	CP025525	903x	This study

								Molecular typing loci						
ID	Year	State	EPS†	MLVA	wgsMLVA	PFGE	Structure	*prn*	*ptxP*	*ptxA*	*fimH*	Accession no.	Depth‡	Reference
I439	2012	CO	Y	27	27	CDC300	Cluster-16	*prn2*-IS*481*-1613fwd	*ptxP3*	*ptxA1*	*fimH1*	CP025375	441x	This study
I462	2012	CT	Y	27	27	CDC253	Cluster-6	*prn2*-IS*481*-240rev	*ptxP3*	*ptxA1*	*fimH1*	CP025376	432x	This study
I598	2013	WA		27	27	CDC010	Cluster-3	*prn2*-promoter_dis	*ptxP3*	*ptxA1*	*fimH1*	CP025380	744x	This study
I602	2013	GA		36	36	CDC237	Cluster-4	*prn2*-IS*481*-1613fwd	*ptxP3*	*ptxA1*	*fimH1*	CP011202	289x	5
I623	2012	VT		27	27	CDC002	Cluster-2	*prn2*-Stop-C1273T	*ptxP3*	*ptxA1*	*fimH1*	CP025386	457x	This study
I692	2011	VT		27	27	CDC002	Cluster-2	*prn2*-Stop-C1273T	*ptxP3*	*ptxA1*	*fimH1*	CP025378	394x	This study
I705	2011	VT		27	27	CDC002	Cluster-2	*prn2*-Stop-C1273T	*ptxP3*	*ptxA1*	*fimH1*	CP025524	113x	This study
I730	2013	CO	Y	27	27	CDC237	Cluster-4	*prn2*-IS*481*-1613fwd	*ptxP3*	*ptxA1*	*fimH1*	CP011203	291x	5
I752	2013	CT	Y	313	313	CDC074	Singleton	*prn2*	*ptxP3*	*ptxA1*	*fimH1*	CP011204	441x	5
I763	2013	VT		27	27	CDC002	Cluster-2	*prn2*-Stop-C1273T	*ptxP3*	*ptxA1*	*fimH1*	CP011205	438x	5
I859	2012	VT		27	27	CDC237	Cluster-4	*prn2*-IS*481*-1613fwd	*ptxP3*	*ptxA1*	*fimH1*	CP025477	197x	This study
I892	2007	VT		16	16	CDC037	Cluster-12	*prn2*	*ptxP3*	*ptxA1*	*fimH2*	CP025479	239x	This study
I896	2007	VT		16	16	CDC170	Cluster-12	*prn2*	*ptxP3*	*ptxA1*	*fimH2*	CP025381	410x	This study
I915	2010	VT		27	27	CDC002	Cluster-2	*prn2*-Stop-C1273T	*ptxP3*	*ptxA1*	*fimH1*	CP011206	293x	5
I944	2013	CA		27	27	CDC104	Cluster-3	*prn2*	*ptxP3*	*ptxA1*	*fimH1*	CP011207	176x	5
I945	2013	GA		27	27	CDC237	Singleton	*prn2*-IS*481*-1613fwd	*ptxP3*	*ptxA1*	*fimH1*	CP025384	206x	This study
I955	2013	CA		27	27	CDC237	Cluster-4	*prn2*-IS*481*-1613fwd	*ptxP3*	*ptxA1*	*fimH1*	CP025531	394x	This study
I958	2013	NM	Y	27	27	CDC237	Cluster-4	*prn2*-IS*481*-1613fwd	*ptxP3*	*ptxA1*	*fimH1*	CP025350	371x	This study
I975	2013	NY	Y	27	27	CDC253	Cluster-6	*prn2*-IS*481*-240rev	*ptxP3*	*ptxA1*	*fimH1*	CP011242	151x	5
I998	2013	WA		27	27	CDC237	Cluster-4	*prn2*-IS*481*-1613fwd	*ptxP3*	*ptxA1*	*fimH1*	CP011243	695x	5
J018	2013	MN	Y	27	27	CDC010	Cluster-3	*prn2*-Stop-C223T	*ptxP3*	*ptxA1*	*fimH1*	CP011208	199x	5
J022	2013	OR	Y	27	27	CDC253	Cluster-6	*prn2*-IS*481*-240rev	*ptxP3*	*ptxA1*	*fimH1*	CP011244	338x	5
J024	2013	NY	Y	27	27	CDC010	Cluster-3	*prn2*	*ptxP3*	*ptxA1*	*fimH1*	CP025353	406x	This study
J038	2013	AZ		27	27	CDC237	Cluster-4	*prn9*-IS*481*-1613fwd	*ptxP3*	*ptxA1*	*fimH1*	CP012087	105x	5
J066	2013	CT	Y	27	27	CDC237	Cluster-4	*prn2*-IS*481*-1613fwd	*ptxP3*	*ptxA1*	*fimH1*	CP026998	329x	This study
J076	2013	NM	Y	27	27	CDC253	Singleton	*prn2*-IS*481*-240rev	*ptxP3*	*ptxA1*	*fimH1*	CP011762	348x	5
J077	2013	MN	Y	27	27	CDC300	Cluster-16	*prn2*-IS*481*-1613fwd	*ptxP3*	*ptxA1*	*fimH1*	CP025344	363x	This study
J085	2013	CO	Y	27	27	CDC375	Singleton	*prn2*-IS*481*-1613fwd	*ptxP3*	*ptxA1*	*fimH1*	CP026997	313x	This study
J139	2013	TX		27	27	CDC377	Singleton	*prn2*-IS*481*-1613fwd	*ptxP3*	*ptxA1*	*fimH1*	CP025527	289x	This study
J184	2012	IN		27	27	CDC002	Cluster-2	*prn2*-Stop-C1273T	*ptxP3*	*ptxA1*	*fimH1*	CP025383	377x	This study
J185	2013	IN		28	28	CDC237	Cluster-4	*prn2*-IS*481*-1613fwd	*ptxP3*	*ptxA1*	*fimH1*	CP025343	344x	This study
J234	2013	VT		27	27	CDC002	Cluster-2	*prn2*-Stop-C1273T	*ptxP3*	*ptxA1*	*fimH1*	CP025348	424x	This study

*EPS, Enhanced Pertussis Surveillance/Emerging Infections Program Network; MLVA, multiple-locus variable number tandem repeat analysis; PFGE, pulsed-field gel electrophoresis; wgs, whole genome sequence.
†Average coverage depth of all PacBio and Illumina sequencing data combined.

Appendix Table 2. Comparison of variable number tandem repeat profiles for H811 isolates of *Bordetella pertussis,* 2000–2013*

Method	MLVA type	VNTR1	VNTR3a	VNTR3b	VNTR4	VNTR5	VNTR6
MLVA	18	8	7	0†	7	6	7
wgsMLVA	27	8	6	7	7	6	7

*MLVA, multiple-locus variable number tandem repeat analysis; VNTR, variable number tandem repeat profile; wgs, whole-genome sequence.
†Isolates collected through the EPS/EIP Network are marked with Y in this column.
‡If 2 discrete bands cannot be observed, traditional MLVA cannot differentiate the 2 VNTR3 loci and VNTR3b is reported as 0.
